# Wood product carbon substitution benefits: a critical review of assumptions

**DOI:** 10.1186/s13021-021-00171-w

**Published:** 2021-03-30

**Authors:** Christina Howard, Caren C. Dymond, Verena C. Griess, Darius Tolkien-Spurr, G. Cornelis van Kooten

**Affiliations:** 1 Canadian Forest Service, Natural Resources Canada, Victoria, Canada; 2 Climate Change and Integrated Planning Branch, Ministry of Forests, Lands, Natural Resource Operations and Rural Development, Government of British Columbia, Victoria, Canada; 3 Institute of Terrestrial Ecosystems, Department of Environmental System Sciences, ETH Zürich, Zurich, Switzerland; 4 Department of Economics, University of Victoria, Victoria, Canada

**Keywords:** Substitution benefit, Displacement factor, Long-lived wood products, Climate change mitigation

## Abstract

**Background:**

There are high estimates of the potential climate change mitigation opportunity of using wood products. A significant part of those estimates depends on long-lived wood products in the construction sector replacing concrete, steel, and other non-renewable goods. Often the climate change mitigation benefits of this substitution are presented and quantified in the form of displacement factors. A displacement factor is numerically quantified as the reduction in emissions achieved per unit of wood used, representing the efficiency of biomass in decreasing greenhouse gas emissions. The substitution benefit for a given wood use scenario is then represented as the estimated change in emissions from baseline in a study’s modelling framework. The purpose of this review is to identify and assess the central economic and technical assumptions underlying forest carbon accounting and life cycle assessments that use displacement factors or similar simple methods.

**Main text:**

Four assumptions in the way displacement factors are employed are analyzed: (1) changes in harvest or production rates will lead to a corresponding change in consumption of wood products, (2) wood building products are substitutable for concrete and steel, (3) the same mix of products could be produced from increased harvest rates, and (4) there are no market responses to increased wood use.

**Conclusions:**

After outlining these assumptions, we conclude suggesting that many studies assessing forest management or products for climate change mitigation depend on a suite of assumptions that the literature either does not support or only partially supports. Therefore, we encourage the research community to develop a more sophisticated model of the building sectors and their products. In the meantime, recognizing these assumptions has allowed us to identify some structural, production, and policy-based changes to the construction industry that could help realize the climate change mitigation potential of wood products.

## Background

It is important to carefully analyze the science informing policy focused on forest carbon strategies for climate change mitigation. In particular, there is interest in the role that forest biomass can play in substituting fossil fuels and non-biomass materials, a great deal of which is associated with the role of long-lived wood products in the construction sector [1–8]. However, analyses of those mitigation strategies depend on assumptions about production levels, economic pricing, markets, and technologies that remain largely untested.

Future atmospheric concentration of carbon dioxide (CO_2_) can potentially be reduced by using wood products in the construction sector. The climate benefits from using wood in construction come from the low fossil fuel energy needed to manufacture wood, the associated circumvention of industrial process emissions related to non-wood product manufacture, the option to use waste wood for bioenergy, and the actual physical carbon stored in wood products [1, 9–12]. These potential carbon benefits are due to the existing structure of the construction and materials sector, which is dominated by products, such as steel, cement, paper, plastics, and aluminum, whose production contributes a large percentage of global anthropogenic carbon emissions [12–14]. For example, production of concrete inherently requires large quantities of material, energy, and water. Concrete production accounts for some seven percent of global CO_2_ emissions, and creates abundant waste at the end of its lifetime [15]. However, there are questions as to whether timber should be left unharvested with carbon stored in situ, or sustainably managed and harvested to supply materials that replace concrete and steel in construction. The climate change mitigation benefit of keeping a forest as a carbon sink or to harvest it depends on several factors, including the inventory and age of standing timber, the growth rate of the forest, the dynamics of the carbon fluxes (including the threat of natural disturbance), the time frame being considered, and the context of carbon displacement factors used when wood products replace non-wood products [16]. This review focuses on the latter issue.

Studies that compare forest management strategies for climate change mitigation may or may not consider product substitution. For those that do, the substitution benefit calculated from the displacement factor can be many multiples of the carbon stored in the forest or in the products themselves [17, 18].

A research paper by Leskinen et al. [12] examines the role of wood use in substituting greenhouse gas intensive-materials and fossil fuels, and subsequently reviews current literature discussing substitution factors. The average substitution effect of the papers reviewed was about 1.2 kg C / kg C, suggesting that every kilogram of C in wood products used to substitute non-wood products resulted in about 1.2 kg C of emissions reduction [12]. The authors also asserted that substitution factors do not provide sufficient information to guide policy making. While useful for greenhouse gas and carbon focused research, this definition is not bound by the market interactions and economic definitions normally associated with the word displacement.

Early work by economists defined a displacement cost in terms of opportunity cost [19]. In the current context, the opportunity cost is measured in terms of carbon fluxes. Thus, if wood products displace concrete and steel in construction, the opportunity cost in terms of carbon (i.e., the displacement cost) is equal to the difference in the carbon flux between using wood and non-wood materials in construction. If concrete and steel lead to greater emissions of greenhouse gases than the wood products that replace them, the displacement factor, measured in terms of CO_2_, would be negative, so that wood material would be preferred to non-wood materials in construction. Following this logic, it also seems important to determine if harvest rates would be modified in response to increased wood use, in order to increase the availability of wood products, or, if the current forest harvest cannot be adjusted, the extent to which wood fiber can be utilized to minimize overall CO_2_ emissions.

Displacement factors are predominantly used to model the carbon benefits of using wood products in place of traditional fossil fuel based products in the construction and energy sectors [12, 20, 21]. It is important to distinguish between the displacement factors in the construction and energy sectors, due to the different assumptions underlying their calculation. Sathre and O’Connor [22] specify that avoided process emissions from manufacturing are one of a number of greenhouse gas related effects considered when using wood building products in place of cement-based products. It remains unclear, however, if the same consideration is commonly given to the process emissions related to fossil fuel production when wood biomass substitutes for fossil fuels in generating electricity, say. It is also important to note that there are seemingly endless combinations of wood products, fossil fuel products, and structural uses that exist in the construction sector, which makes determining a conclusive displacement factor for a given scenario complex. For example, Harmon [23] indicates that, when laminated beams are produced rather than sawn softwoods (lumber), some 63 to 83% more energy is required, leading to a lower displacement factor.

These considerations lead to a general stratification of existing literature, where most publications will focus on either the construction or the energy sector, with some overlap in studies that account for the bioenergy potential of buildings at end-of-life. Within the forestry industry, there is some overlap between these two sectors. For example, sawmill residual waste could be used to produce an engineered wood product, pulp or energy. Therefore, an increase in demand for one product could cause a change in the availability of another product.

**Fig. 1:**
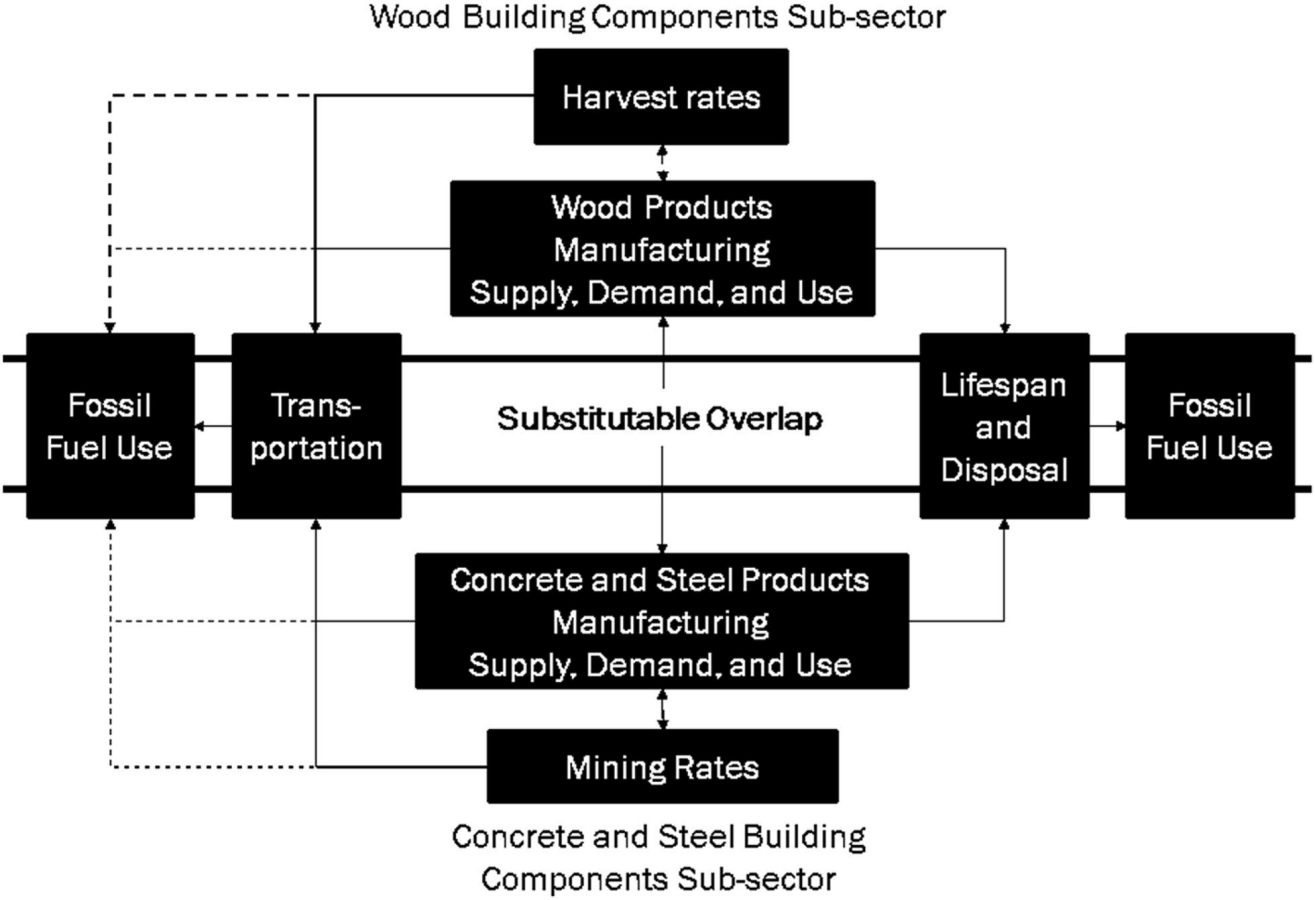
Building sector economic structure, as implied by a full lifecycle analysis. Arrows indicate dependencies

The purpose of this review is to identify and assess the economic and technical assumptions in the forest carbon accounting assessments that use displacement factors. This will be done by following the methodology of Schlamadinger and Marland [24], or as commonly used in attributional Life Cycle Assessments (LCA) [25–28]. We are primarily concerned with the building sector, although a similar review could be done for the energy sector. Figure 1 illustrates the building sector’s economic market structure in terms of wood products, as implied by the methods under review. The assumptions we have identified for testing are as follows: (1) Changes in harvest or production rates will lead to a corresponding change in wood product consumption, as well as an opposite response in concrete, steel, or fossil fuel use. (2) Wood building products are substitutable for concrete and steel. (3) The same mix of products could be produced from increased harvest rates. (4) There are no market responses to increased wood use. As an illustration, we investigate whether these assumptions are reflective of current realities within the Canadian forest industry and wood product manufacturing. In the following sections, we examine the assumptions in detail.

This inquiry is important due to the increasing recognition of wood substitution as a sustainable environmental contribution and important climate change mitigation strategy and the large benefits conveyed by product displacement [17, 29, 30]. Further, it builds on the recent sensitivity analysis by Harmon [23], who found that wood product substitution benefits might be overestimated by 2 to 100%. Our literature-based investigation of a number of different assumptions underlying the calculation of displacement benefits will help to determine the likelihood that the related substitution benefits are either too optimistic or too pessimistic. Validating the methodology used to calculate displacement benefits will help to determine what needs to be done from a policy perspective to achieve a higher level of substitution, given current characteristics of the Canadian construction sector. Improved understanding and tools may be needed to better account for the intricacies that are required for the realistic application of displacement factors.

## Main text

### Assumption 1

*“Changes in harvest or production rates will lead to a corresponding change in wood product consumption, as well as an opposite response in concrete, steel, or fossil fuel use.”*

There is a basic assumption imbedded in the use of displacement factors, namely, that increasing the supply of primary wood will increase the overall consumption of wood products [24, 31]. Economic theory tells us that an increase in supply of primary wood causes the wood supply function to intersect the demand function at a lower price, when other factors affecting demand for wood products remain unchanged, which leads to increasing purchases. However, the extent to which the lower price results in greater use of wood products in construction is more nuanced as it depends on the elasticities of supply and demand, where the wood is consumed, or whether it is stored or burned. For example, smaller logs are used to manufacture a different mix of forest products if harvest rates on a fixed site are increased. Thus, it remains a challenge to establish that increases in harvest rates cause changes in consumption of wood products. Changes in harvests and production are also impacted by regulatory and trade policies, such as the U.S.-Canada Softwood Lumber Agreement [32]. Such policies are not necessarily accompanied by a corresponding increase or decrease in the consumption of particular wood products. Some studies indicate that if the demand for wood products increases, the price of these products will increase, stimulating increased supply, harvest, global export of those products [33]. For example, Johnston and van Kooten [34] found that European subsidies for wood pellets that replace coal in generating electricity could cause substantial disruptions in global forest product markets. This would increase the prices of wood pellets, pulp wood and oriented strand board and similar products, while decreasing the price of lumber.Even with some studies showing linkages between harvest and consumption, many model-based findings report an expected increase or decrease in harvest rates, but many of those do not report a corresponding increase or decrease in wood product consumption, as is assumed in substitution calculations. For example, increasing forest conservation areas or forest species’ protection can cause domestic harvest rates to decrease, but can increase harvest rates in other geographic areas if the conservation policy leads to increases in prices [35–37]. This is referred to as a leakage [28]. In this context, there are many papers that discuss the high leakage rates related to harvest shifting in the forest industry [38–40]. However, it is unclear how increased rates of leakage will affect the production of specific wood products, and therefore, how leakage will affect consumption of these wood products. Papers discussing the possible leakage rates of the concrete and steel industries in reaction to an increasing market share of wood products in the construction sector are difficult to find, suggesting that empirically determined leakage rates regarding price and elasticity are somewhat absent. This absence contributes to the difficulty of determining how increased harvest may or may not be directly linked with increased consumption of wood products in the construction sector.

Harvest rates do not directly influence wood product consumption, but harvest rates do seem to shift and influence each other on a global scale through price changes. For example, while global wood use has remained steady since 1990, wood use has declined in developed countries and risen in developing countries, with global roundwood production increasingly coming from illegal logging activities [29]. While much of this harvest in developing countries is going towards fuelwood, it is likely that any pulp or paper produced from wood harvested in developing countries could further reduce North American timber harvests [41], however, with an undetermined effect on consumption of these products. Outside of real-world examples, some model-based publications have estimated the link between harvest rates and wood product consumption. Upton et al. [42] included an assumption within a modeling framework, that when concrete is assumed to be the main building material in the US construction sector in the model, the surplus forest that is no longer harvested for construction materials still undergoes a leakage of approximately 20%. This assumes that demand for forest products does not completely control the harvest rates in U.S. forests, due to the assumption that forests are not perfectly managed. Similarly, Eriksson et al. [1] suggest that an increase in wood construction will have only a minor impact on harvests, achieved through the balance of increased sawlog harvest and decreased pulpwood harvest.

In the Canadian context, it would be beneficial to determine the rate of forest harvest leakage, or potential for leakage, in the domestic forest sector. A study by Gan and McCarl [43] investigated international leakage rates resulting from the implementation of more forest conservation, finding that Canada is at low risk of high leakage compared to other countries/regions considered important in the world’s production, consumption, and trade of forest products. Consequently, Canada would be a good place to implement more forest conservation areas that store carbon [43]. This is an artefact of Canada’s tenure system and policies. The vast majority of Canada’s forests are publicly owned and inaccessible to economic exploitation, while provincial governments regulate harvest rates. Given that most forests are beyond the extensive margin (inaccessible to forest operations), a domestic push to increase the amount of long-lived wood products in Canada would need to be associated with policies that increase forest harvest utilization at the intensive margin. This, however, would also lead to greater investments in silviculture and CO_2_ emissions associated with such investments. Since wood products tend to have lower CO_2_ emissions associated with them, we are not going to recommend policies to reduce wood product leakage.

While there is some evidence that increased harvests will increase consumption of wood products, there are limited studies available that show this relationship empirically given historic data. In conclusion, it may be important to show this linear relationship in future studies focused on harvest rates and resulting effects on consumption, allowing policy intended to take advantage of this relationship to have a positive result, in terms of reduced emissions.

### Assumption 2

*“Wood building products are substitutable for concrete and steel.”*

Wood products are likely a better option than steel or concrete in the building sector, in terms of climate change mitigation and reduced emissions [40]. While many wood products are currently used in the construction sector, it is unclear how directly substitutable all possible products or wood-based designs are for the current typical building design. Largely, the substitutability is taken into account in the studies underlying the displacement factors that compare functionally equivalent buildings or building elements [44, 45]. Factors influencing substitutability include technical properties of wood products as compared to concrete and steel, price of these new wood products as compared with traditional building materials, acceptance of new building materials and building codes, regulation of new wood products, and education of industry related stakeholders. Further, at the end of their lifetime, wood building products generally need to be used to create bioenergy that displaces fossil-fuel energy sources, which provides a further impetus to their displacement benefit [45]. A study by Nässén et al. [46] even suggested that wood-based buildings would only be of benefit when the bioenergy produced from the end-of-life products was generated with carbon capture and storage technology capabilities.

A wood-frame building that consists primarily of joist beams [47] was considered to be directly substitutable for current reinforced concrete buildings in Italian areas, and cross-laminated timber is increasingly entering the construction sector in taller and taller buildings [48]. However, Hurmekoski et al. [49] suggest that most building types and blueprints will have to be changed in order for the share of wood products to increase in the construction sector. Thus, most building codes would have to be changed to accommodate adding more wood products into the construction market [29]. If this were the case, further investigation into how policy or blueprint changes could affect a community’s housing capacity would be needed. An updated version of the National Building Code of Canada (NBC) in 2015 allowed for the construction of wood framed structures up to six storeys, and Natural Resources Canada reports that this has resulted in over 500 mid-rise buildings to be completed, under construction, or in the design and development stage. Displacement factors are assumed to provide functional equivalents to existing end use products; therefore, the functional equivalency of a wood building as compared to a concrete building may need to be further examined in future studies.

Price can also be a barrier to substitution of wood for concrete and steel. Engineered wood products, such as wood fiber insulation boards, cross-laminated timber, laminated veneer lumber and glulams, can have matching technical properties to building materials that rely more on fossil fuels in their production. However, they are not currently economically capable of providing anything more than niche products in a larger construction industry [50]. Guardigli et al. [47] suggests that wood may not be able to penetrate the construction sector until technology is economically competitive with existing building materials. Some possible solutions to allow wood building materials to become economically competitive include a carbon tax on products, subsidies, or procurement policies, such as supporting first use. Overall, policy instruments that allow for the external costs of carbon emissions to be internalized will provide a structural change that could increase the use of wood products [51].

Other market characteristics need to be considered. For example, adoption of new wood technologies is dependent on the diffusion of manufacturing technology into the risk-averse construction industry [49]. Concerning tall wood buildings, steel and concrete are both 150 year-old industries [52]. It is difficult to determine if new and emerging wood product technologies will saturate the construction industry. Due to the current resiliency of the construction system, the lack of knowledge within the industry about applications of wood products, the lack of financing, insufficient incentives for replacing old technology, and high costs, the increase of wood products in the industry is less likely and hindered [23, 50].

From a market-demand perspective, it may be important to determine whether there is a negative attitude towards using more long-lived wood products in buildings. A survey of support for forest carbon mitigation strategies found that individuals either directly or indirectly employed by the B.C. forestry sector were less likely to support any of the proposed mitigation strategies [53]. However, it is likely that increasing the production of longer-lived wood products through enhanced forest management could be of direct benefit to the B.C. forestry sector [53]. Therefore, to increase forest sector support for wood building construction, it may be important to help spread awareness of the positive economic effects that this enhanced forest management may have on the employment opportunities within the sector.

In terms of the Canadian public, there is a lack of studies directly analyzing general perceptions of engineered wood products; however, some papers have examined the public attitude toward long-lived wood products within other geographic areas. The majority of respondents to a survey within the US Pacific Northwest said that tall wood buildings had greater fire risk, required more maintenance, and were not as durable as steel or concrete [54]. Interview data from the UK construction sector indicate that end-users with a lack of information on wood products are prejudiced against the use of wood as a building material, as they believe it has inferior fire resistance and inherent safety issues [55]. If these data are applied to the Canadian construction sector, it may be important to increase public education on the safety and benefits of wood products, or there may be a lack of public approval of wood building construction.

Architects will also play an important role in the adoption of increased construction of engineered wood product buildings. Within Sweden, a survey sent to architects found a few common reasons for not selecting engineered wood products for buildings, including not being the one in charge of making material choices, not having enough knowledge about the materials, and uncertainty about the quality of the materials’ appearances or durability over time [56]. However, they also found that reasons for architects to select engineered wood products included a perceived low impact on the environment and aesthetics [56]. To increase architect approval and use of wood products within their designs, it may be important to focus mainly on these factors for selection, and increase the amount of information available surrounding the factors for avoidance.

An example of long-lived wood product use in Canada was realized with the construction of the Brock Commons building on the campus of the University of British Columbia in Vancouver. Brock Commons is an 18-storey residential hybrid structure, constructed with a combination of concrete, steel, cross-laminated timber (CLT), glue-laminated timber (GLT), and parallel strand lumber (PSL) [57]. The Teshnizi et al. [57] study shows that Brock Commons performs better than a conventional reinforced concrete structure in a number of environmental impact categories, including global warming potential and fossil fuel depletion potential. However, the study also finds that the total cost of ownership associated with the structures is higher, by about 7% per square meter [23]. A limitation of the Brock Hall study was the lack of available data concerning certain context-specific environmental information, which suggests that more data would be necessary in order for decision-makers to rely on these or similar results in planning future projects. However, they also point out that acquiring much of this data can be time consuming, and that it may not be feasible to collect during the design phase of future buildings. Even with these limitations, if the cost of tall wood buildings remains more expensive than conventional buildings, price may be a barrier to increasing the use of CLT, GLT and/or PSL in the future.

Capacity to produce wood products can also be a barrier to substitution. In Canada, mass-timber buildings are commonly constructed from CLT engineered wood products, due to their high quality and stability characteristics [58]. As an example, CLT was one of the primary wood products used in constructing the Brock Commons building at UBC [59]. While demand for Canadian softwood lumber and structural panels is expected to increase in response to growing North American housing markets, there is a chance that Canadian production may be negatively affected by pest infestations and forest fires in Western Canada [60]. Given this potential decline in Canadian capacity to produce CLT, a policy-mandated increase in uptake of engineered wood products would need to consider the sources of available CLT, as this may make Canada more dependent on engineered wood products from other countries.

In conclusion, the challenges associated with adopting wood use in the current construction industry is important to understand before using displacement factor values to inform policy. Authors of existing studies may understand that a displacement factor is based on a counterfactual analysis of increased wood in construction. However, the assumptions underlying that counterfactual scenario need to be understood by policy makers intending to increase wood products for the intention of increased avoided emissions.

### Assumption 3

*“The same mix of products could be produced from increased harvest rates of a given area.”*

In some of the climate change mitigation analyses, there is an expectation that forest harvest or utilization will increase in order to provide additional long-lived wood products [61, 62]. However, few published papers provide evidence as to whether the kind of forest that is harvested will subsequently change, or how homogenous the forest resource is. It is important to understand what proportion of forest harvest is going to short-lived or long-lived wood products. For example, in Germany, about 47% of annual timber harvest is going towards short-lived products with an average lifetime below 25 years, while only 22% of annual timber harvest was used as construction wood with an average lifetime of about 50 years [63]. For British Columbia, the proportion of long-lived wood products was about 1/3 with half-lives ranging from 30 to 90 years [64, 65]. This information is relevant, as not all forest types can be used to create long-lived products; considerations need to be made concerning tree species, timber diameter, quality of carbon storage, and thinning requirements before one can assume that a particular stand of trees is suitable for the construction sector [63]. For example, sawlog and pulpwood harvests are not directly substitutable, since an increase in production of sawlogs tends to increase the production of pulpwood, but an increase in production of pulpwood tends to decrease the production of sawlogs [35, 66]. In order to make more forests applicable for long-lived wood product production, rotation times must be increased [39], basal area must be increased [16], and overall quality of the wood produced must be evaluated [1].

Within Canada, forest carbon modelling projects assess a number of different individual strategies for climate change mitigation. Many studies have investigated both better harvest utilization and longer-lived wood products as forest management strategies for climate change mitigation purposes [61, 62, 67]. The higher utilization strategy increases the merchantable utilization and salvage harvesting, effectively increasing the percentage of stemwood transferred to wood products without changing the total area of harvest. This assumption relies on some of the statements discussed above, in that the same forest area is able to provide increased harvest volume that is of at least equal quality to the original harvested wood. The longer-lived wood product strategy increases the proportion of harvested wood going towards products like panels and away from pulp and paper. Similarly, this relies on the assumption that the wood being used to produce the pulp and paper is just as suitable for the construction of solid wood products. Further, both the increased utilization and the longer-lived wood product strategies can be combined together to produce additive interactions, as greater harvests become available for producing more long-lived products. Higher utilization and longer-lived wood products are often found to be one of the best forest management pathways for reducing emissions within these studies, so it would be beneficial to determine how Canada can implement policy to support these actions.

In this context, it may be important to understand how lower quality or smaller trees can be used to create engineered wood products, especially products like CLT. One study found that, although sawing smaller diameter logs normally produces a lower volume yield than larger diameter logs, a live sawing and trapeze-edging method for CLT panel production would increase yield by almost 20%, compared to a business as usual cutting method [68]. Another study by Espinoza and Buehlmann [69] suggests that underutilized, low-value, and disturbance affected hardwood species may be excellent options for producing CLT. However, there are technical and policy barriers to these options for producing CLT from smaller diameter or lower quality trees. It is important that the forest industry is able to provide the necessary technical guidance for the mass production of these products, and there needs to be an internal push to start prioritizing the harvest of underutilized hardwood versus softwood species, even with existing price differences [68]. The possibility exists that hybrid CLT could be made, but this still requires technical guidance from industry.

To support these actions, there may need to be government subsidies or programs that allow and encourage forest managers and industry members to pursue alternative wood product production, which will help relieve the economic pressure that could be a barrier to producing engineered wood products. A wide-sweeping carbon tax may increase the value of harvest residuals for products, especially if business as usual is to burn these residues, as is common in British Columbia. Research and development focused on increasing the use wood products will be more useful if a range of products is studied, including existing and in-development product types. Both utilization standards and secondary fibre access tenures can help increase access to unused forest residue for licensees and stakeholders. It may become increasingly important to require companies and licensees to prove that they have tried to sell any remaining harvest residues before giving a burn permit. Ultimately, modifying harvest levels will likely need to be achieved through strong legal commitment to sustainable forest management, thereby retaining extant carbon stored in forests [70].

In conclusion, the homogeneity of the forest resource included within a substitution factor needs to be considered. If a policy maker wishes to realize the substitution benefit suggested within a research paper, they need to ensure that the forest resource in question can easily be used to create the product intended. If this assumption is not considered, the capacity to create a product may not exist in the forest resource, resulting in the calculated displacement factor to be incorrect. For example, more demand for CLT could take lumber away from the single family homes, resulting in little or no displacement benefit, unless the CLT is made from fibre used for shorter lived products.

### Assumption 4

*“There are no market responses to increased wood use.”*

Here we need to distinguish between two different perspectives: the first is that of a typical attributional life-cycle analysis (LCA) where an economic agent (consumer) must choose between two different products. Once the choice is made, the other product is not consumed nor created and, thus, those emissions do not occur [71]. The second perspective is that of the atmosphere. If a policy is put in place, or a product is consumed, do the emissions from the alternate policy or product still occur? For example, suppose a policy change means that more multi-family dwellings are constructed out of wood instead of concrete and steel. From the builder’s perspective, the substitution has occurred, but, from the perspective of the atmosphere, it follows that, if demand for multi-family buildings made of concrete and steel goes down, the subsequent fall in the price of steel and concrete would thereby increase the use of such materials elsewhere, leading to cross-sectoral leakage [34]. We have not been able to find studies on cross-sectoral leakages in the construction industry. However, economists have found evidence of carbon leakages more generally when only a subset of jurisdictions impose climate mitigation policies; emissions are simply moved to different jurisdictions. Worse, the net effect can potentially result in a green paradox where total emissions increase [72, 73], although this rarely is the case in the real world. Harmon [23] found that potential substitution benefits were highly sensitive to cross-sectoral leakage rates.

Potentially, cross-sectoral leakage could be considered in accounting processes by assuming that emissions are avoided until the end of the period under consideration, or by assuming avoidance due to the development of alternative technologies [74]. A common methodological issue related to this non-permanence is that storage for 100 or more years is considered permanent [75]. The only way to guarantee emission avoidance is to either produce a new technology that outcompetes higher CO_2_ products, or produce emissions today that sequester in a permanent sink, both of which seem unlikely [75]. However, more issues need to be addressed somewhat more closely.

From a Canadian perspective, it may be necessary to examine the policy options for dealing with the cross-sectoral leakage. It would also be important to define the length of time associated with permanence in Canada, as noted. A common methodological timeline to adopt is about 100 years [75]. However, a more appropriate and useful approach, due to Ciricacy-Wantrup [76], is to weight physical carbon fluxes as to when they occur. This makes the choice of an appropriate weighting scheme (essentially a rate for discounting future carbon fluxes) a policy choice that is determined by the urgency associated with the need to address climate change [28, 77]. Suppose a tree-planting project removes 1000 tCO_2_ from the atmosphere 101 years from now. With a 100-year time-line, the carbon removed in year 101 is irrelevant. Thus, this policy implicitly assumes carbon is discounted at an annual rate of about 15%; this rate would make removals in year 101 or later effectively equal zero today. A severe climate emergency would suggest that CO_2_ removed from the atmosphere after 20 years is irrelevant as society no longer exists as we know it, then a removal of 1000 tCO_2_ in year 21 has to be discounted at an annual rate of more than 90% to make it irrelevant today. The rate chosen to weight future carbon is a policy instrument that depends on the urgency with which climate change must be mitigated [34]. Considering these policy relevant discounting options may help limit the cross-sectoral leakage that could decrease the ability to fully realize suggested substitution benefits.

If a 100-year timeline is chosen, it may be important to investigate the likelihood that the wood building products are used to generate electricity, say, with associated emissions released after that 100-year timeline is reached. This also requires an understanding of future energy intensities and likely technological developments in carbon capture, as the re-remission of carbon may be less of a concern if the emission intensities of construction products have been lowered overall. Carbon capture technologies are quickly developing to provide oil and gas industries the option to remain profitable under increasingly stringent emissions reduction goals. However, the cost of getting carbon capture technologies wrong or over-estimating their current capacity for capture could have lasting negative effects from a climate change mitigation perspective [78]. We could find no examples of carbon capture pertaining to building element manufacturing.

Other approaches to reducing cross-sectoral leakage include an economy-wide carbon tax and jurisdictions committing to global action to reduce CO_2_e emissions. We expect markets to react such that if demand for concrete and steel building products goes down, their price would fall. A carbon tax on such products would reduce the appeal, and hopefully reduce the occurrence of cross-sectoral leakage. However, these actions work best when distributed globally across international markets. Hence the recommendation for commitment internationally to climate change mitigation targets.

## Conclusions

Many studies assessing forest management or products for climate change mitigation depend on a suite of assumptions that the literature either does not support or only partially supports. Ignoring or misunderstanding these assumptions could result in decreased actualized avoided emissions, when compared to the original displacement factor suggested in a research paper. Understanding these potential consequences of the assumptions underlying this methodology is important if society is successfully to implement structural production and policy-based changes to the construction industry. Changes to the carbon accounting or life cycle systems could include adding both harvest shifting and cross-sectoral leakage. An economic model representing price and market characteristics could be used to help define more sophisticated displacement factors for a range of situations. Both carbon and economic studies should also recognize implicit or explicit technological assumptions. Governments could help reduce the uncertainty caused by the assumptions reviewed above by committing to global climate action that includes the forest sector, applying carbon taxes to products with higher global warming emissions, and implementing programs to reduce cultural, educational, and technological barriers to substitution.

## Data Availability

Not applicable.

## Abbreviations

CO_2_: Carbon dioxide
LCA: Life cycle assessment
NBC: National building code of Canada
CLT: Cross laminated timber
GLT: Glue laminated timber
PSL: Parallel strand lumber
